# *Mycobacterium avium* Subspecies *paratuberculosis* Recombinant Proteins Modulate Antimycobacterial Functions of Bovine Macrophages

**DOI:** 10.1371/journal.pone.0128966

**Published:** 2015-06-15

**Authors:** John P. Bannantine, Judith R. Stabel, Elizabeth Laws, Maria Clara D. Cardieri, Cleverson D. Souza

**Affiliations:** 1 National Animal Disease Center, USDA-Agricultural Research Service, Ames, Iowa, United States of America; 2 Department of Veterinary Clinical Sciences, Washington State University, Pullman, Washington, United States of America; University of Minnesota, UNITED STATES

## Abstract

It has been shown that *Mycobacterium avium* subspecies *paratuberculosis* (*M*. *paratuberculosis*) activates the Mitogen Activated Protein Kinase (MAPK) p38 pathway, yet it is unclear which components of *M*. *paratuberculosis* are involved in the process. Therefore, a set of 42 *M*. *paratuberculosis* recombinant proteins expressed from coding sequences annotated as lipoproteins were screened for their ability to induce IL-10 expression, an indicator of MAPKp38 activation, in bovine monocyte-derived macrophages. A recombinant lipoprotein, designated as MAP3837c, was among a group of 6 proteins that strongly induced IL-10 gene transcription in bovine macrophages, averaging a 3.1-fold increase compared to non-stimulated macrophages. However, a parallel increase in expression of IL-12 and TNF-α was only observed in macrophages exposed to a subset of these 6 proteins. Selected recombinant proteins were further analyzed for their ability to enhance survival of *M*. *avium* within bovine macrophages as measured by recovered viable bacteria and nitrite production. All 6 IL-10 inducing MAP recombinant proteins along with *M*. *paratuberculosis* cells significantly enhanced phosphorylation of MAPK-p38 in bovine macrophages. Although these proteins are likely not post translationally lipidated in *E*. *coli* and thus is a limitation in this study, these results form the foundation of how the protein component of the lipoprotein interacts with the immune system. Collectively, these data reveal *M*. *paratuberculosis* proteins that might play a role in MAPK-p38 pathway activation and hence in survival of this organism within bovine macrophages.

## Introduction

*Mycobacterium avium* subsp. *paratuberculosis* (*M*. *paratuberculosis*) is a pathogen with a broad host-range characterized by the capacity to evade the immune system and to cause severe chronic intestinal granulomatous inflammation. Although it can infect multiple species, it is primarily a disease problem in ruminants, which include cattle, sheep and goats [1]. The lack of a complete understanding of the host immune response against this pathogen has hindered the development of an effective vaccine. A sustained research effort has been focused on the biology of *M*. *paratuberculosis* to improve our knowledge and understanding of the infection process [1, 2]. The dairy industry incurs substantial economic losses due to reduced milk production, premature culling and reduced slaughter value [3]. The bacterium is shed in the feces and milk of infected animals, primarily in the clinical phase of disease [4]. Transmission of disease can occur by ingestion of the bacterium through manure-contaminated feedstuffs and pastures or by colostrum and milk, passed from the infected dam to the calf [4, 5].

Pathogenic mycobacteria interfere with the phagosome maturation process, but the precise mechanism has not been fully detailed [6]. *M*. *paratuberculosis* is known to survive within macrophages by impairing nitric oxide production [7] and has been shown to resist physiological concentrations of nitric oxide [8]. However, *M*. *paratuberculosis* appears susceptible to IFN-γ induced activation of cultured macrophages which supports the fact that elevated levels of IFN-γ are present in cows with subclinical Johne’s disease and the cytokine appears important for controlling mycobacterial infection [9]. Nonetheless, addition of IFN-γ to *M*. *paratuberculosis*-infected macrophage cultures does not appear to promote killing of the bacteria [7, 8, 10]. Another mechanism by which *M*. *paratuberculosis* survives within macrophages is by inhibiting phagosome acidification and maturation. Phagosomes containing *M*. *paratuberculosis* do not accumulate lysosomal markers and do not acidify lower than pH 6.3 in J774 macrophages [11], indicating a failure of the phagosome to mature into a phagolysosome.

Previous studies showed that *M*. *paratuberculosis* infection of bovine macrophages results in increased IL-10 transcription and decreased IL-12 transcription [12, 13], a gene expression pattern that promotes interaction with the innate immune receptor Toll-like receptor 2 (TLR2) and activation of intracellular immune cytokine regulator, MAPKp38, in bovine macrophages [14, 15]. Mitogen Activated Protein kinases (MAPK) are stress activated kinases with the MAPKp38 kinase existing as four isoforms, alpha, beta, gamma and delta [16]. In the case of p38 alpha, it is the sites Thr180/Tyr182 that become dual phosphorylated, signaling activation within the cell. MAPKp38 is activated as a result of cellular stresses, most notably the presence of inflammatory cytokines [16]. Because the MAPKp38 pathway is a mechanism for suppression of antimicrobial responses within macrophages, activation of this pathway could enable intracellular survival of *M*. *paratuberculosis*. It also induces production of the anti-inflammatory cytokine interleukin (IL)-10 [13, 14]. Induction of IL-10 has been described as playing key roles in dampening the immune system, favoring *M*. *paratuberculosis* survival within host cells [17].

Conversely, tumor necrosis factor (TNF)-α is produced by macrophages and dendritic cells as a primary response to infections and tissue damage [18]. TNF-αplays an important role in activation and recruitment of leukocytes to inflamed tissue [18], and has been demonstrated to be involved in the host-defense against *M*. *tuberculosis* [19]. However, TNF-α is also associated with excessive inflammation and immunopathology in infections and autoimmune diseases. The specific role of the MAPKp38 pathway in phagosome maturation during mycobacterial infection is not completely understood; however, it has been shown that *M*. *paratuberculosis* infection of cultured bovine macrophages results in a rapid phosphorylation of MAPKp38 [14]. The pathogenic pathway that initiates with *M*. *paratuberculosis*-TLR2 engagement leading to activation of the MAPKp38 pathway and culminating with high levels of IL-10 production can also be exploited to rationally design a critically needed vaccine against *M*. *paratuberculosis*.

Lipoproteins are involved in a variety of functions including cell wall synthesis, adhesion, transmembrane signaling and anchoring proteins to the cell membrane [20]. It is known that mycobacterial lipoproteins are recognized by TLR [21]. Mannosylated lipoarabinomannan (Man-LAM) is a mannose-capped lipoglycan cell wall component of pathogenic mycobacteria that was previously shown to induce strong expression of IL-10 [22]. Since Man-LAM contains lipid and lipoproteins are part of a larger group of microbial molecules that are called pathogen-associated molecular patterns (PAMPs) that interact with TLRs [23–25], we reasoned that lipoproteins may be involved in MAPKp38 activation. Furthermore, two lipoproteins that result in attenuation of *M*. *tuberculosis* when disrupted include LpqS [26] and LspA [25]. Therefore, the *M*. *paratuberculosis* genome was searched for all genes annotated as lipoproteins and they were expressed and purified from *E*. *coli* and used to test activation of MAPKp38, ability to stimulate expression of IL-10 and its capacity to prevent killing of *M*. *avium* subspecies *avium (M*. *avium)* by bovine macrophages. At least two recombinant proteins were discovered that have modular effects on macrophage-mycobacterial interactions. Genes encoding these recombinant proteins are considered targets for constructing directed knockout mutations to test attenuation in bovine macrophages.

## Materials and Methods

### Monocyte isolation and macrophage generation

All work involving animals was conducted in accordance with the recommendations in the institutional guidelines and approved animal care and use committee (IACUC) protocols at Washington State University. All other experiments were carried out in accordance with the Washington State Universities’ Institutional Biosafety Committee (IBC) approved protocol number 1190 along with and the National Animal Disease Center’s IBC-0261 protocol.

Blood samples used for isolation of monocytes were collected from three healthy adult Holstein dairy cows that tested negative for paratuberculosis as determined by culture and IS900 PCR analysis of fecal samples. Peripheral blood mononuclear cells were isolated by centrifugation on a Percoll density gradient as described [12]. Briefly, blood was layered onto 50 mL conical tubes containing Histopaque 1077 (Sigma-Aldrich, USA), and following density gradient centrifugation (500 x g for 20 minutes) at room temperature, peripheral blood mononuclear cells (PBMC) were collected. Thereafter, PBMCs were washed twice with sterile phosphate-buffered saline (PBS; Invitrogen, Life Technologies, USA) before resuspending cells in PBS. Monocytes were then isolated using microbeads conjugated with mouse anti-human CD14 antibody (isotype mouse IgG2a; Miltenyi Biotec Ltd., San Diego, USA), which has been shown to be cross-reactive with bovine monocytes [27]. The isolation was performed according to the manufacturers’ instructions. The identity and purity of monocytes (>97%) was determined by flow cytometry using an anti-CD14 fluorescein-labeled antibody (data not shown). Purified monocytes were seeded at 2x10^6^ per well in 12-well tissue culture plates containing Dulbecco's Modified Eagle medium with high glucose (Invitrogen, Life Technologies, USA) with 10% heat inactivated fetal calf serum (Sigma-Aldrich, USA), gentamicin (5 mg/ml; Sigma-Aldrich, USA), 100 ng/mL GM-CSF (Kingfisher, USA) and 1mM β-mercaptoethanol (Sigma-Aldrich, USA). Subsequently, cells were incubated at 37°C in a 5% CO_2_ humidified atmosphere. On day 7 confluent macrophages were used in all described experiments.

### Culture conditions for *Mycobacterium avium* subspecies

*M*. *paratuberculosis* K-10 and *M*. *avium* subspecies *avium* strain ATCC 35716, originally isolated from cattle, was obtained from the American Type Culture Collection, USA. *M*. *avium* cells were grown to a concentration of approximately 10^8^ CFU/ml, washed, and resuspended in Middlebrook broth containing Oleic Albumin Dextrose Catalase Growth Supplement, and Tween 80. Viability of the organisms added to macrophage cultures varied between 85% and 95% as determined by propidium iodide exclusion (data not shown). Immediately before addition to macrophage cultures, organisms were washed in warm PBS and resuspended in RPMI1640 medium without antibiotics. *M*. *paratuberculosis* was cultured only for the MAPK-p38 activation experiment.

### Production and purification of recombinant *M*. *paratuberculosis* proteins

*M*. *paratuberculosis* genes annotated as lipoproteins were selected for cloning into the pMAL-c2x expression vector and transformed into *E*. *coli* DH5α. All clones were confirmed to be correct and in-frame with the maltose binding protein by DNA sequencing. To obtain the MBP-lacZ alpha peptide control protein, the native pMAL-c2x vector, without a cloned insert, was expressed in the same way as the *M*. *paratuberculosis* recombinant clones. Confirmed transformants were cultured, induced with IPTG and recombinant fusion proteins purified as described previously [28]. The only modification was that proteins eluted off the amylose resin column were collected and loaded onto a second amylose column to maximize removal of potential LPS contamination. LPS contamination was ruled out for a selection of these proteins using the Limulus amebocyte lysate gel clot assay (Lonza).

### Infection and RNA extraction of cultured macrophages

*M*. *avium* organisms (MOI: 10 bacilli/macrophage) were added to cell cultures with and without addition of *M*. *paratuberculosis* recombinant proteins (5 ug/ml) and incubation was continued at 37°C in 5% CO_2._ Cellular mRNA was harvested from plates at 2 hours using the RNeasy kit (Qiagen, USA) following the manufacturer instructions. The RNA purity was assessed by measuring the 260/280 ratio with Nanodrop (Nanodrop Products, Wilmington, USA). Integrity of RNA preparations was assessed by use of RNA agarose gel electrophoresis. As a control for DNA contamination, a direct PCR was performed to confirm the absence of β-actin amplification in RNA samples. RNA samples were stored in 10 μL aliquots at -80°C until further processing.

### Determination of cytokine gene expression by quantitative real time-PCR

Genomic DNA was removed from mRNA samples by use of a commercial kit (RNeasy plus Mini Kit, Qiagen, USA) following the manufacturer instructions immediately after mRNA isolation from cultured macrophages. First-strand cDNA was synthesized by use of a commercial kit (Script cDNA Synthesis Kit, Bio-Rad, USA) following the manufacturer instructions. Then, cDNA was diluted to 100 μl total volume and SYBR green master mix was added (Power SYBR Master Mix, Life Technologies, USA). Samples were analyzed in triplicate in a 96-well optical reaction plate. Each sample contained 5 μl of cDNA diluted to 1:10 in DNAse free water and 15 μl of SYBR green master mix. Primers (Table 1) were designed using a web-based program; http://biotools.umassmed.edu/bioapps/primer3_www.cgi). Gene expression was evaluated as relative fold expression using the ∆∆Ct method. GAPDH was used as an endogenous control to normalize the gene expression input. Preliminary results showed no variation in the expression of GAPDH in macrophages treated with the chemical MAPKp38 inhibitor (SB203580, Sigma-Aldrich, USA), or DMSO to untreated macrophages (data not shown).

**Table 1:** Primers used for qRT-PCR.

Gene	Primer	Accession no.
TNF-α	Forward 5′– TCAAACACTCAGGTCCTCTTCTCA––3′ Reverse 5′ - GTCGGCTACAACGTGGGCTACC––3′	AC000180
IL-10	Forward 5′–CGGCTGCGGCGCTGTCATC–3′ Reverse 5′–TCACCTTCTCCACCGCCTTGCTCT–3′	P43480
IL-12 (p40)	Forward 5–TCGGCAGGTGGAGGTCA–3 Reverse 5–ACACAAAACGTCAGGGAGAAGTAG–3	P46282
GAPDH	Forward 5′–GAAACCTGCCAAGTATTGATGAGAT–3′ Reverse 5′–TGTAGCCTAGAATGCCCTTGAGAG–3′	P10096

### Determination of nitric oxide production

After 24-hour incubation of *M*. *paratuberculosis*-derived peptides (5 μg/mL) with primary bovine macrophages, nitrite (i.e., the stable by-product of nitric oxide generated by phagocytes) was measured in culture supernatants. Fifty microliters of supernatant was mixed with 200 μL of Griess reagent (1% sulfanilamide, 0.1% napthylethylenedamine dihydrochloride, and 2.5% H_3_PO_4_) and incubated at 25°C for 10 minutes. Absorbance was determined at 540 nm, and absorbance readings were converted to micromolar concentrations by comparing results for samples with results for a standard curve generated by use of concentrations ranging from 1.5 to 200 μM of NaNO_2_.

### Phagocytosis and intracellular survival of mycobacterial organisms

*M*. *avium* organisms were used instead of *M*. *paratuberculosis* in the survival assay because macrophages have been shown to more effectively kill *M*. *avium* organisms therefore increasing the assay sensitivity. Monocyte-derived macrophages attached to coverslips were stained with Ziehl-Neelsen carbolfuchsin stain (Sigma, St Louis, MO) for presence of mycobacteria and other acid-fast organisms. The percentage of macrophages containing organisms was determined by counting a minimum of 200 cells by use of light microscopy. Killing of organisms was assessed by use of a live-dead stain (BackLight kit, Invitrogen, Carlsbad, CA). This test has previously been reported to provide a rapid and reliable method for differentiating live vs. dead *M*. *avium* organisms [14]. Macrophages were preincubated with or without *M*. *paratuberculosis*-derived recombinant proteins (5 μg/mL) for 2 hours and then infected with *M*. *avium*. After 72 hours, macrophages were washed twice in PBS solution and then lysed by incubation with 0.1% deoxycholate for 5 minutes. The lysate was incubated with a 1:1 mixture of a green fluorescent stain and propidium iodine stain. Cells were placed on a microscope slide, cover-slipped, and examined on a fluorescent microscope (40X objective) by using a dual-band filter set that detects fluorescence in the green and red emission spectra. For this method, live organisms had green fluorescence and dead organisms had red fluorescence. At least 200 organisms were enumerated per treatment group.

### Determination of MAPKp38 phosphorylation by enzyme-linked immunosorbent assay (ELISA)

An ELISA kit (InstantOne, eBioscience, USA) was used to measure phosphorylated levels of MAPKp38α in bovine macrophage lysates post treatment with either ovalbumin (OVA, Life Technology, USA) at 5 ug/mL, live *M*. *paratuberculosis* organisms at 10:1 MOI or *M*. *paratuberculosis* recombinant proteins at 5 ug/mL. Resting macrophages were used as the background control. A modified protocol was used such that 300 μL cell lysates were added to completely cover a single well in a standard 12-well plate. Equal parts cell lysate plus capture and detection antibody reagents were added simultaneously to the InstantOne assay plate. After 1 hour of incubation at 37°C, the wells were washed and detection solution (supplied by the kit) was applied for 20 minutes in the dark. Absorbance was measured at 450 nm in a standard ELISA plate reader. Positive control cell lysate and negative control (cell lysis buffer), as well as untreated lysates, confirmed antibody efficacy. Experimental replicates were done in triplicate.

### Statistical analysis

All tests were performed in triplicate and results of at least three separate experiments were evaluated. Mann-Whitney statistical test was used to verify normal distribution of the data. Results were expressed as mean ± SD. Differences between cell cultures incubated with *M*. *paratuberculosis* recombinant proteins with or without adding *M*. *avium* were analyzed by use of the paired student t-test. *P< 0*.*05* was considered to be statistically significant.

## Results

### *M*. *paratuberculosis* recombinant proteins stimulate cytokine transcription

**Fig 1:**
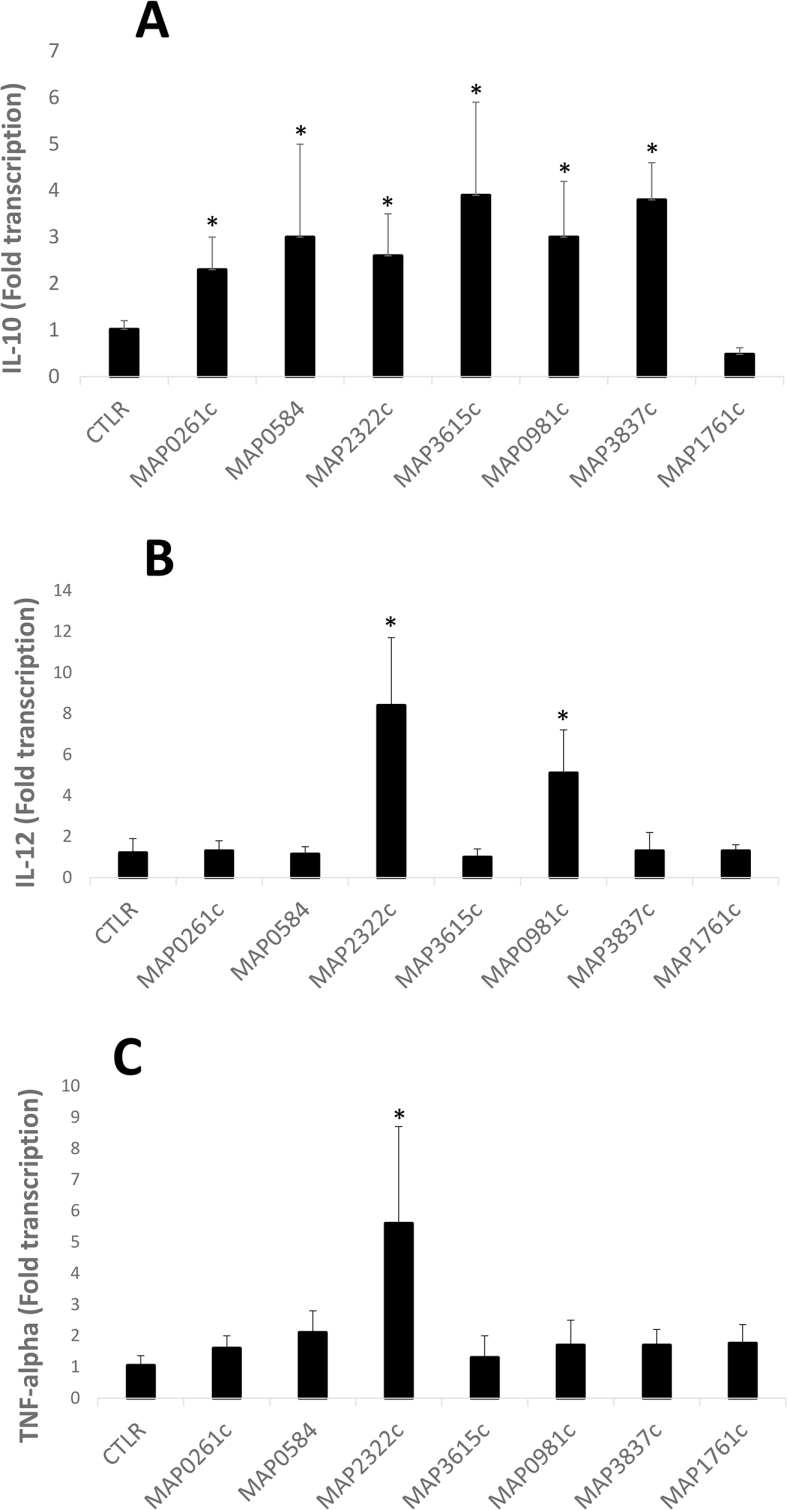
Cytokine expression levels of bovine macrophages in response to MAP recombinant proteins. Shown are IL-10 (A), IL-12 (B) and TNF-α (C), mRNA transcription levels in response to 2-h incubation with the recombinant proteins indicated on the x-axis. Significantly different macrophages treated with MAP lipoproteins in comparison to macrophages alone (CTLR) are indicated with an asterisk (*). Error bars represent results of at least three separate experiments.

Since it is known that the MAPKp38 pathway induces expression of IL-10, while at the same time suppressing IL-12 expression, we tested transcription of these cytokines in macrophages after exposure to *M*. *paratuberculosis* recombinant proteins. It is further known that lipid containing molecules such as the 19-kDa antigen and trehalose 6, 6’-dimycolate activate the TLR2 pathway [24, 29], thus we reasoned that *M*. *paratuberculosis* lipoproteins may also be involved in TLR2 interactions. Therefore the genome sequence of *M*. *paratuberculosis* [30] was searched for genes annotated as lipoproteins. A total of 51 genes satisfied these criteria. Of these, 42 genes (82%) were successfully cloned and expressed in *E*. *coli* (Table 2). Macrophages were incubated with or without these purified fusion proteins and analyzed for cytokine expression including TNF-α, IL-12 and IL-10 transcription (Table 2). The results show 6 of 42 proteins had greater than 2 fold increase of IL-10 transcription over background. These six include MAP0261c, MAP0584, MAP2322c, MAP3615c, MAP0981c and MAP3837c (Fig 1A). MAP2322c also stimulated transcription of both IL-12 and TNF-α above the level of the control, whereas MAP0981c stimulated transcription of only IL-12 (Fig 1B and 1C). These data initially suggest MAP0261c, MAP0584 and MAP3837c are the primary candidates for stimulating MAPKp38 phosphorylation.

**Table 2:** *Mycobacterium avium* subspecies *paratuberculosis* protein effect on NO2 and cytokines.

		Conc.	NO2	TNF-α		IL-12		IL-10	
Protein	Description	(A550)	(ug/cc)	fold change	SD	fold change	SD	fold change	SD
**MAP0261c**	19-kDa lipoprotein, LpqH	0.048	2.7	1.527	0.498	1.318	0.492	**2.255**	0.7
**MAP0584**	Lipoprotein, LprH	0.067	3.8	2.11	0.749	1.132	0.366	**3.044**	2.045
MAP2009	Zn-dependent hydrolase	0.04	2.3	3.182	1.951	1.04	0.854	1.208	0.242
MAP3268	Small heat shock protein	0.036	2.0	0.885	0.223	0.56	0.175	0.187	0.068
MAP1138c	Lipoprotein, LprG	0.051	2.9	1.826	0.465	2.231	0.651	1.806	0.478
MAP0474c	Lipoprotein, LpqE	0.09	5.1	1.067	0.372	0.103	0.046	1.072	0.387
MAP2048	Lipoprotein, LppO	0.083	4.7	1.414	0.695	0.052	0.024	0.449	0.243
MAP2522	Lipofamily protein, LprE	0.065	3.7	1.155	0.578	1.032	0.256	0.361	0.064
MAP3883c	beta-lactamase protein	0.049	2.8	3.08	1.711	2.097	0.957	0.325	0.069
MAP1194c	27-kDa lipoprotein	0.07	4.0	3.401	0.268	2.122	0.935	1.187	0.243
MAP3417c	Lipoprotein, LpqC	0.048	2.7	2.655	1.573	1.117	0.549	1.039	0.314
MAP0466c	Lipoprotein, LpqF	0.104	5.9	1.079	0.473	0.308	0.101	1.078	0.404
MAP2017	Lipoprotein, LppN	0.077	4.4	0.625	0.251	0.331	0.149	0.761	0.356
MAP2498c	Lipoprotein, LprB	0.063	3.6	1.185	0.636	1.041	0.288	0.728	0.197
MAP3688	beta-glucosidase, LpqI	0.078	4.4	1.191	0.66	1.047	0.539	0.756	0.388
MAP0989	Hypothetical protein, LpqU	0.084	4.8	1.459	1.062	1.06	0.353	0.828	0.387
MAP1840	Lipoprotein, LppK	0.067	3.8	7.671	2.796	3.267	1.502	1.315	0.55
MAP0440c	Hypothetical protein, LpqG	0.09	5.1	2.914	1.792	1.476	1.061	1.517	1.178
MAP1909	Lipoprotein, LppM	0.075	4.2	0.06	0.028	0.928	0.587	1.471	0.966
**MAP2322c**	Hypothetical protein, LppS	0.096	5.4	5.688	3.152	8.496	3.37	**2.625**	0.939
**MAP3615c**	Hypothetical protein, LprO	0.083	4.7	1.274	0.79	1.09	0.433	**3.913**	2.026
**MAP0981c**	Lpp-LpqN family protein	0.086	4.9	1.698	0.823	5.149	2.193	**3.047**	1.225
MAP2216c	Lipoprotein, LppR	0.047	2.7	1.016	0.181	1.012	0.154	1.38	0.95
MAP3056	pknH-like protein, LpqA	0.065	3.7	8.09	5.651	9.111	5.045	0.555	0.255
MAP1761c	peptidase M75 protein	0.043	2.4	1.761	0.602	1.29	0.314	0.48	0.139
MAP1781	Lipoprotein, LppI	0.036	2.0	3.984	0.483	3.593	1.672	0.639	0.2
MAP0670	D-ala-D-ala dipeptidase, LpqR	0.036	2.0	0.781	0.508	0.663	0.399	0.982	0.188
MAP2103c	LppP/LprE lipoprotein family	0.049	2.8	3.364	1.737	2.761	1.937	1.764	1.088
MAP3481	Histidine phosphatase, LpqD	0.037	2.1	1.049	0.317	1.058	0.346	1.175	0.616
MAP3041	Dehydrogenase, LppZ	0.057	3.2	1.47	0.364	1.436	0.496	0.539	0.373
MAP3908	Lipoprotein peptidase, LpqM	0.044	2.5	1.033	0.259	1.022	0.209	0.42	0.172
MAP1670c	L, D-transpeptidase, LppS	0.062	3.5	3.9	2.122	3.251	1.855	0.628	0.344
**MAP3837c**	Hypothetical protein, LpqJ	0.062	3.5	1.627	0.534	1.288	0.886	**3.863**	0.818
MAP1604c	19-kDa lipoantigen, LppE	0.053	3.0	2.893	1.02	3.826	1.35	1.723	0.736
MAP2497c	Lipoprotein, LprC	0.065	3.7	1.864	0.821	2.165	0.806	1.158	0.585
MAP2548c	Solute binding protein, LpqY	0.063	3.6	2.469	1.359	3.666	1.754	0.699	0.257
MAP3906	Amidohydrolase, LpqL	0.049	2.8	1.607	1.258	1.104	0.467	0.329	0.085
MAP2417c	Hypothetical protein, LppJ	0.043	2.4	2.773	1.078	0.738	0.291	0.303	0.138
MAP2539c	Substrate binding protein, LpqZ	0.043	2.4	1.592	1.239	1.163	0.594	0.338	0.177
MAP3907	Peptidase M28, LpqL	0.064	3.6	0.428	0.395	3.145	1.817	1.335	0.442
MAP1216c	Lipoprotein, LpqQ	0.069	3.9	0.136	0.062	3.064	1.576	1.134	0.585
MAP1397	Hypothetical protein, LprJ	0.056	3.2	0.138	0.096	1.401	1.046	1.02	0.257
LacZ	Non-mycobacterial control	0.08	4.5	1.542	0.415	0.579	0.27	1.076	0.398

### MAP1761c increases survival of *Mycobacterium avium* in macrophages

**Fig 2:**
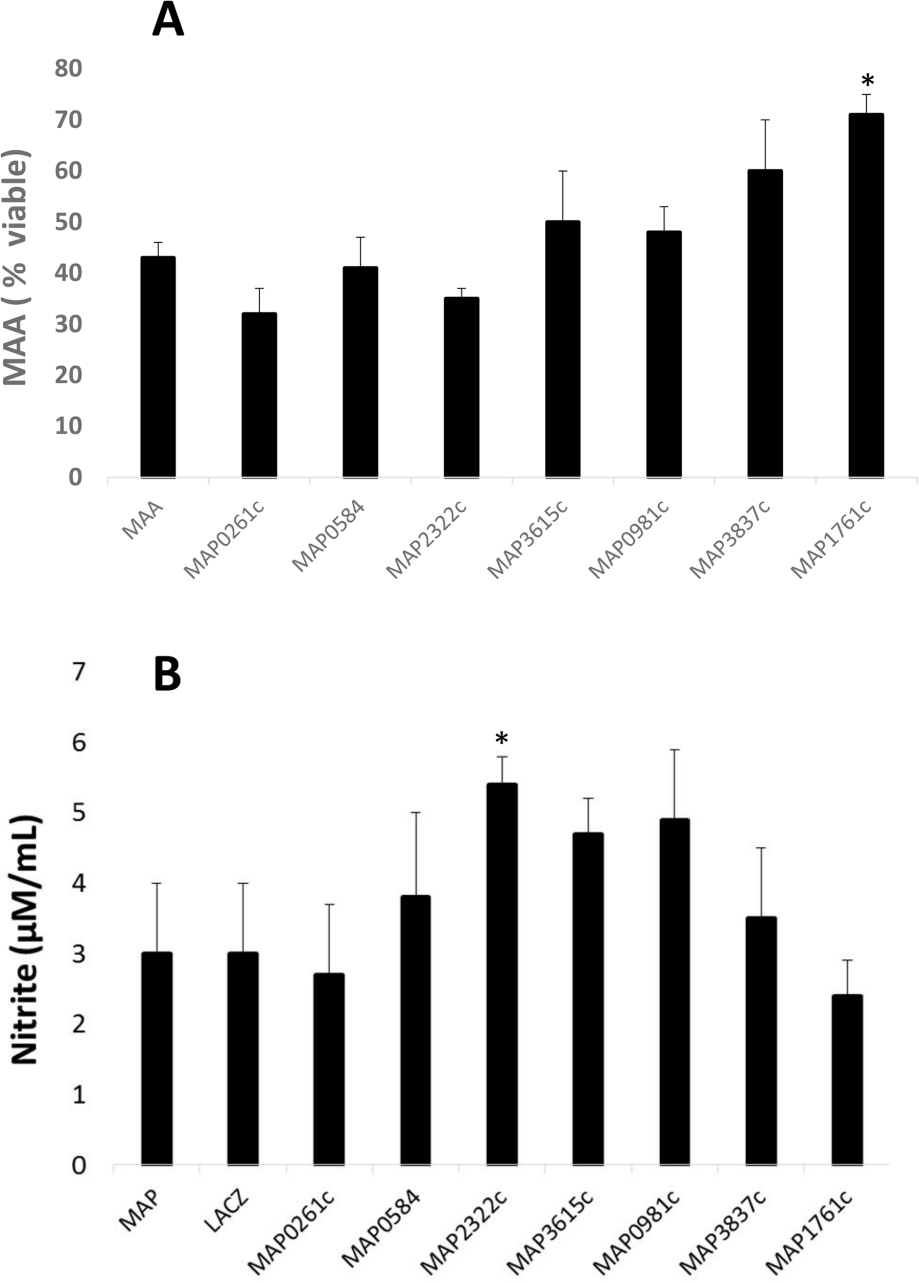
MAP1761c promotes survival of *M*. *avium* in bovine macrophages. Macrophages were incubated with or without recombinant proteins (5 μg/ml) for 2 h and then infected with *M*. *avium* for 72 h. (A) Preincubation of macrophages with MAP1761c resulted in 71±4% viability. The other lipoproteins shown were either at or below the percent viability for *M*. *avium* cells alone. (B) Only MAP2322c induced significant levels of nitrite production compared to macrophages incubated with MAP and LacZ. Error bars represent results of at least three separate experiments.

Although it has been shown that some *M*. *avium* strains can survive within human and murine macrophages [31], other studies have shown that *M*. *avium* is more susceptible to killing within cultured bovine macrophages than is *M*. *paratuberculosis* [10, 32]. Therefore, to evaluate the effect of *M*. *paratuberculosis* proteins on *M*. *avium* survival within bovine macrophages, cells were preincubated in the presence or absence of recombinant proteins for 2 hours and then incubated with *M*. *avium* for 72 hours, which is the time needed to kill approximately half of the *M*. *avium* inoculum [10]. When macrophages were exposed to MAP1761c, *M*. *avium* survival was enhanced with 71±4% of the cells surviving after 72 hours (Fig 2A). MAP3837c seemed to also have a preservation effect as 60±10% of the inoculum survived in macrophages exposed to that protein. In contrast, only 32±5% *M*. *avium* cells survived after preincubation with MAP0261c (Fig 2A).

### MAP2322c increases production of nitric oxide

Nitric oxide (NO) is a reactive signaling molecule and an important inflammatory mediator, which acts as a cytotoxic agent in addition to modulate immune responses and inflammation through multiple immune networks. Our results showed that compared to macrophages incubated with *M*. *paratuberculosis* and the control peptide LacZ, only MAP2322c shows significant increase in production of NO (Fig 2B).

### The MAPKp38 pathway is activated by *M*. *paratuberculosis* proteins

**Fig 3:**
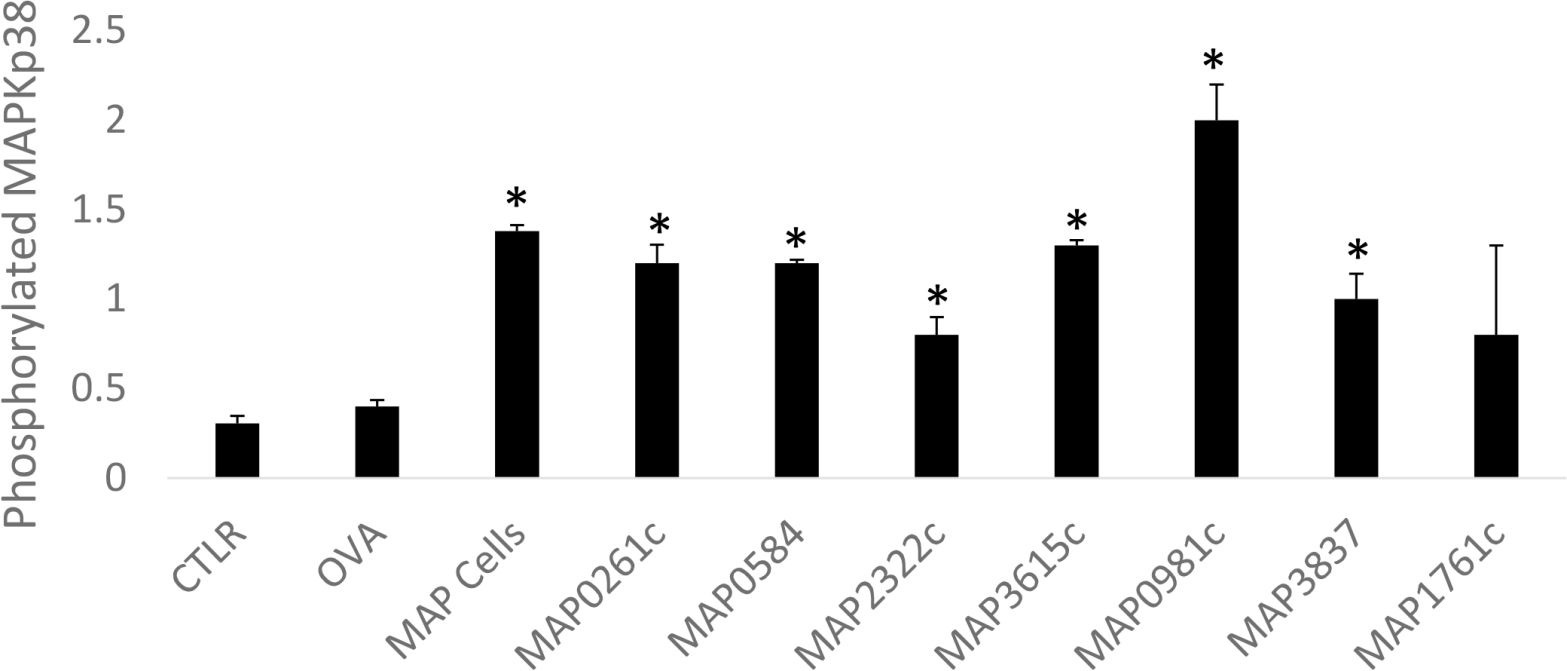
Phosphorylation of MAPK-p38 indicates activation of this pathway by MAP lipoproteins. Bovine macrophages exposed to *M*. *paratuberculosis* cells as well as recombinant proteins showed significant phosphorylation of MAPK-p38 compared to resting macrophages or macrophages incubated with a control peptide ovalbumin (OVA). Significantly different macrophages treated with purified proteins in comparison to macrophages alone (CTLR) are indicated with an asterisk (*).Error bars represent results of at least three separate experiments.

The effects of *M*. *paratuberculosis* recombinant proteins on phosphorylation of MAPKp38 in bovine macrophages were investigated. Compared to macrophages alone or macrophages incubated with OVA, recombinant proteins as well as live *M*. *paratuberculosis* cells significantly enhanced phosphorylation of MAPKp38 (Fig 3). However, only MAP0981c stimulated phosphorylation at a higher level than *M*. *paratuberculosis* cells. These recombinant proteins consistently and reproducibly activated MAPKp38. In contrast, MAPKp38 phosphorylation was attenuated after incubation of macrophages with MAP1761c (data not shown) a protein that failed to induce IL-10 expression (Table 2).

## Discussion

Previous studies have suggested that Man-LAM purified from *M*. *paratuberculosis* may interact with mannose receptors on macrophages to promote IL-10 production and inhibit phagosome acidification [22]. This glycolipid complex was not shown to activate the MAPKp38 pathway, but it is known that *M*. *paratuberculosis* itself activates this pathway [14]. In the present study, we identified specific *M*. *paratuberculosis* proteins that consistently activate MAPKp38 in a manner similar to *M*. *paratuberculosis* whole cells. Although Man-LAM demonstrated a much higher fold increase (15-fold) in IL-10 expression [22] than did the proteins tested here, MAP0584 and MAP3837c did show over 3-fold increase in IL-10 expression by macrophages. MAP1761c did not induce expression of IL-10 (Table 2), but appears similar to MAP3837c in that it appears to inhibit killing of *M*. *avium* within macrophages, although MAP3837c was not statistically significant Inhibition of killing by these proteins are independent of NO production (Fig 2B). MAP1761c is a predicted periplasmic lipoprotein involved in iron transport and contains the peptidase M75 motif on the C-terminal half. This motif was shown to contain proteolytic activity in *Pseudomonas aeruginosa* although its active site residues have yet to be defined [33]. MAP3837c is annotated as a hypothetical lipoprotein so it is unclear what specific role it may play in the bacterial cell, particularly after engagement with the host macrophage, but its participation in MAPKp38 signaling is apparent.

Our previous data suggest IL-10 is a mediator of *M*. *paratuberculosis* survival in macrophages and may suggest the TLR2-MAPKp38 signaling pathways are involved in suppression of bacterial killing [15]. We suspect that TLR2 is a key receptor that interacts with *M*. *paratuberculosis* in some way to prevent it from being killed. Thus, demonstrating which *M*. *paratuberculosis* protein molecule interacts with TLR2 is a critical first step. Although Man-LAM induces a lengthy IL-10 response [22], preliminary studies suggest it does not interact with TLR2. Unfortunately, demonstration of the lipoproteins and their interaction with TLR2 are more difficult to evaluate. Dose and time are critical for each protein and for this assay lipidated proteins are essential. Future studies include testing MAPKp38 phosphorylation after exposure to MAP1761c and MAP3837c. This will include Western blotting with and without the addition of anti-TLR2 antibody for blocking. In addition, an alternative approach that can be pursued to verify the interaction of the proteins studied here with TLR2 is to use the HEK-Blue Detection Kit (Invivogen) that is designed to provide a sensitive and reliable method to screen and validate TLR agonists

It should be noted that because the recombinant proteins were expressed in *E*. *coli*, it is unlikely they were post-translationally lipidated. While all bacteria have the machinery to lipidate proteins, *E*. *coli* is generally viewed as not suitable for producing lipid-modified mycobacterial proteins. Purification of this extensive set of proteins from the *M*. *paratuberculosis* host in quantities that could be analyzed was not feasible. Other groups have been successful in producing recombinant forms of the *M*. *tuberculosis* lipoproteins LppX and LprF using the surrogate host *M*. *smegmatis* for structural studies [34, 35]. This is the strategy we will pursue with a selection of the best candidates identified in the current study. Nonetheless, with this limitation in mind, we can still make inferences regarding the protein component of the lipoprotein and its immune stimulatory capabilities. However, we acknowledge it is likely the effects observed using these recombinant proteins would either be enhanced or unexpectedly altered if using the native lipidated protein.

Others have shown that IL-12 transcription is increased in *M*. *paratuberculosis* infected macrophages within 6 hours and this expression remains high through 24 hours but then decreases to background levels by 72 hours post infection [10]. This suggests that *M*. *paratuberculosis*-infected macrophages rapidly produce IL-12 to enhance the developing T cell response. As shown in Table 2, a set of recombinant proteins consistently increased the transcription of IL-12 and TNF-α but had little effect on IL-10 transcription. Moreover, these proteins failed to activate MAPKp38. It would be reasonable to assume that *M*. *paratuberculosis* contains a cohort of lipoproteins with pro-immune effects but that this is counterbalanced by the presence of anti-inflammatory and immunomodulatory lipoproteins. The net effect is represented by the capacity of this pathogen to circumvent the antimicrobial functions of macrophages. Importantly, immunostimulatory lipoproteins with little effect on MAPKp38 activation could be used to attempt the synthesis of unit-particle vaccines using nanotechnology approaches.

In conclusion, we showed that specific *M*. *paratuberculosis* recombinant proteins induce expression of IL-10 by macrophages whereas others are involved in preserving mycobacteria within macrophages as well as activating the MAPKp38 pathway. These data suggest that *M*. *paratuberculosis* proteins described herein are potentially major virulence factors and that *M*. *paratuberculosis* somehow actively modulates the MAPKp38 signaling pathway.
